# Imaging methods for surgical revascularization in patients with moyamoya disease: an updated review

**DOI:** 10.1007/s10143-021-01596-0

**Published:** 2021-08-21

**Authors:** Lanxin Du, Hanyu Jiang, Jin Li, Ting Duan, Chenyun Zhou, Feng Yan

**Affiliations:** 1 Department of Ultrasound & Key Laboratory of Birth Defects and Related Diseases of Women and Children of Ministry of Education, West China Second University Hospital, Sichuan University, 20#, Section 3, Renmin South Road, 610041 Chengdu, Sichuan People’s Republic of China; 2 Department of Ultrasound, West China Hospital of Sichuan University, 37 Guoxue Street, 610041 Chengdu, Sichuan People’s Republic of China; 3 Department of Radiology, West China Hospital of Sichuan University, 37 Guoxue Street, 610041 Chengdu, Sichuan People’s Republic of China; 4 Department of Neurosurgery, West China Hospital of Sichuan University, 37 Guoxue Street, 610041 Chengdu, Sichuan People’s Republic of China; 5 Clinical Ultrasound Imaging Drug Research Lab, West China Hospital of Sichuan University, 37 Guoxue Street, 610041 Chengdu, Sichuan People’s Republic of China

**Keywords:** Moyamoya disease, Revascularization, Neuroimaging, Angiography, Ultrasound

## Abstract

Neuroimaging is crucial in moyamoya disease (MMD) for neurosurgeons, during pre-surgical planning and intraoperative navigation not only to maximize the success rate of surgery, but also to minimize postsurgical neurological deficits in patients. This is a review of recent literatures which updates the clinical use of imaging methods in the morphological and hemodynamic assessment of surgical revascularization in patients with MMD. We aimed to assist surgeons in assessing the status of moyamoya vessels, selecting bypass arteries, and monitoring postoperative cerebral perfusion through the latest imaging technology.

## Introduction

Moyamoya disease (MMD) is an uncommon cerebrovascular disease characterized by progressive stenosis of the terminal portion of the internal carotid artery (ICA) and its main branches [37]. The disease is associated with the development of dilated, fragile collateral vessels, termed as moyamoya vessels (MMVs). The hemorrhagic and ischemic types are the two main clinical manifestations. The diagnosis of MMD mainly depends on neurological symptoms and imaging findings. Neurosurgical revascularization is considered the mainstay treatment in symptomatic patients to increase intracranial cerebral blood flow (CBF) and cerebrovascular reserve (CVR) [40]. The stenotic degree of the ICA, the compensatory ability of the collateral circulation, the selection of bypass area and the matching of bypass vessels, and the monitoring of postoperative cerebral perfusion are the key points that surgeons care about, which cannot be separated from the support of neuroimaging. In recent years, the concepts of “flow-controlled bypass” and “precised bypass” have been put forward, aiming to improve the cerebral perfusion in preoperatively ischemic areas, reduce the ineffective bypass, and reduce the cerebral hyper-perfusion syndrome (CHS) caused by excessive bypass.

In this review, we will provide an update on the morphological and hemodynamic assessment of common neuroimaging techniques used in surgical revascularization in MMD and introduce other emerging imaging methods, such as indocyanine green video-angiography and ultrasonography, which are simple and practical for intraoperative assessment. This review compares the benefits and drawbacks of various imaging techniques in the perioperative period of MMD from different perspectives, so as to provide a reference for the selection of surgeons, in order to provide the success rate of revascularization and obtain satisfactory long-term outcomes.

## Method

The PubMed, Ovid, Embase, and Cochrane databases were searched over a 20-year period between 2001 to 2021 using the Boolean search term (“moyamoya disease” OR “moyamoya syndrome” OR “MMD”) AND (“revascularization” OR “bypass surgery” OR “STA-MCA bypass” OR “direct surgery” OR “indirect surgery”) AND (“imaging” OR “Computed tomography” OR “Digital subtraction angiography” OR “Magnetic resonance imaging” OR “Single-photon emission computed tomography” OR “Positron emission tomography” OR “Fluorescence imaging” OR “Ultrasonography”). References of each manuscript were checked for papers that were of potential relevance to our review. Two authors (D.L.X. and Y.F.) independently identified articles using the above search criteria. Relevant articles on the application of imaging technologies in moyamoya disease in the past decade were mainly included. For the decade 2001–2011, high-quality articles highly relevant to the content of this review were mainly included. The full text of each selected article was obtained and analyzed.

### Digital subtraction angiography (DSA)

**Fig. 1:**
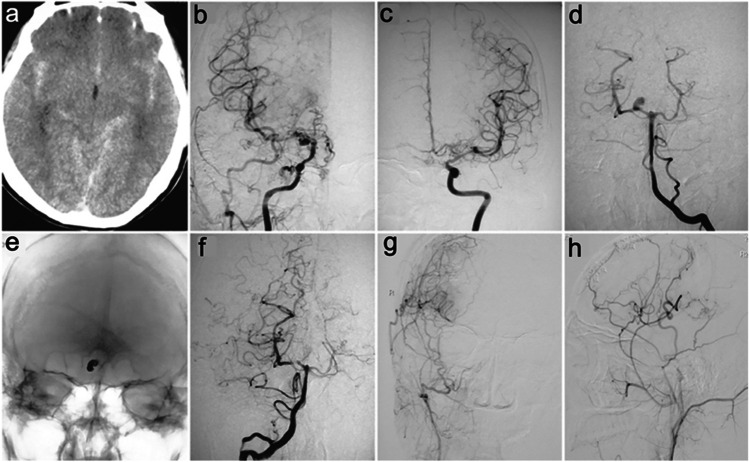
A 40-year-old MMD patient with severe explosive headache was found to have a saccular aneurysm by DSA. The aneurysm was occluded by coil embolization immediately after DSA. Right STA-MCA bypass combined with EDMS was performed 1 month after SAH ictus. **a** CT scan revealed SAH in the longitudinal fissure, Sylvian fissure, and quadrigeminal cistern. **b** and **c** Bilateral ICA angiography studies showing MMD with Suzuki Stage III. **d** Vertebral angiography study showing a saccular aneurysm at the P1/P2 junction of the right PCA. **e** and **f** Follow-up vertebral angiography studies showing complete obliteration of the aneurysm. **g** and **h** ECA angiography studies showing Matsushima Grade A collateral compensation supplied by the anastomosis [50]

DSA is considered the gold standard in diagnosing MMD [44]. Owing to its high spatial and temporal resolution, this method plays an irreplaceable role in assessing the steno-occlusion of the terminal ICA, and the patency of anastomosis. Moreover, DSA is the best choice to observe the establishment of collateral circulation, which is crucial for the decision of treatment strategies and the evaluation of the neo-angiogenesis status after surgery [14, 15, 43]. Compared with other imaging modalities, DSA has superior diagnostic value for detecting concomitant diseases such as intracranial aneurysm (Fig. 1), arteriovenous malformation, and subarachnoid hemorrhage in patients with MMD [4, 42, 50].

Quantitative color-coded parametric DSA (QDSA, Syngo iFlow) is an emerging DSA technique which provides objective perfusion parameters like time-to-peak (TTP), mean transit time (MTT), and ratios of area under the curve (AUC ratio) to assess hemodynamic changes of a certain artery after bypass surgery. Although it does not provide information on cerebral parenchyma perfusion, it is useful for measuring blood supply from the internal and external carotid artery systems [8, 9].

However, DSA is not recommended for pediatric patients or patients in poor conditions, due to its invasiveness, time consumption, and need for anesthesia.

### Computed tomography

#### Computed tomography angiography (CTA)

**Fig. 2:**
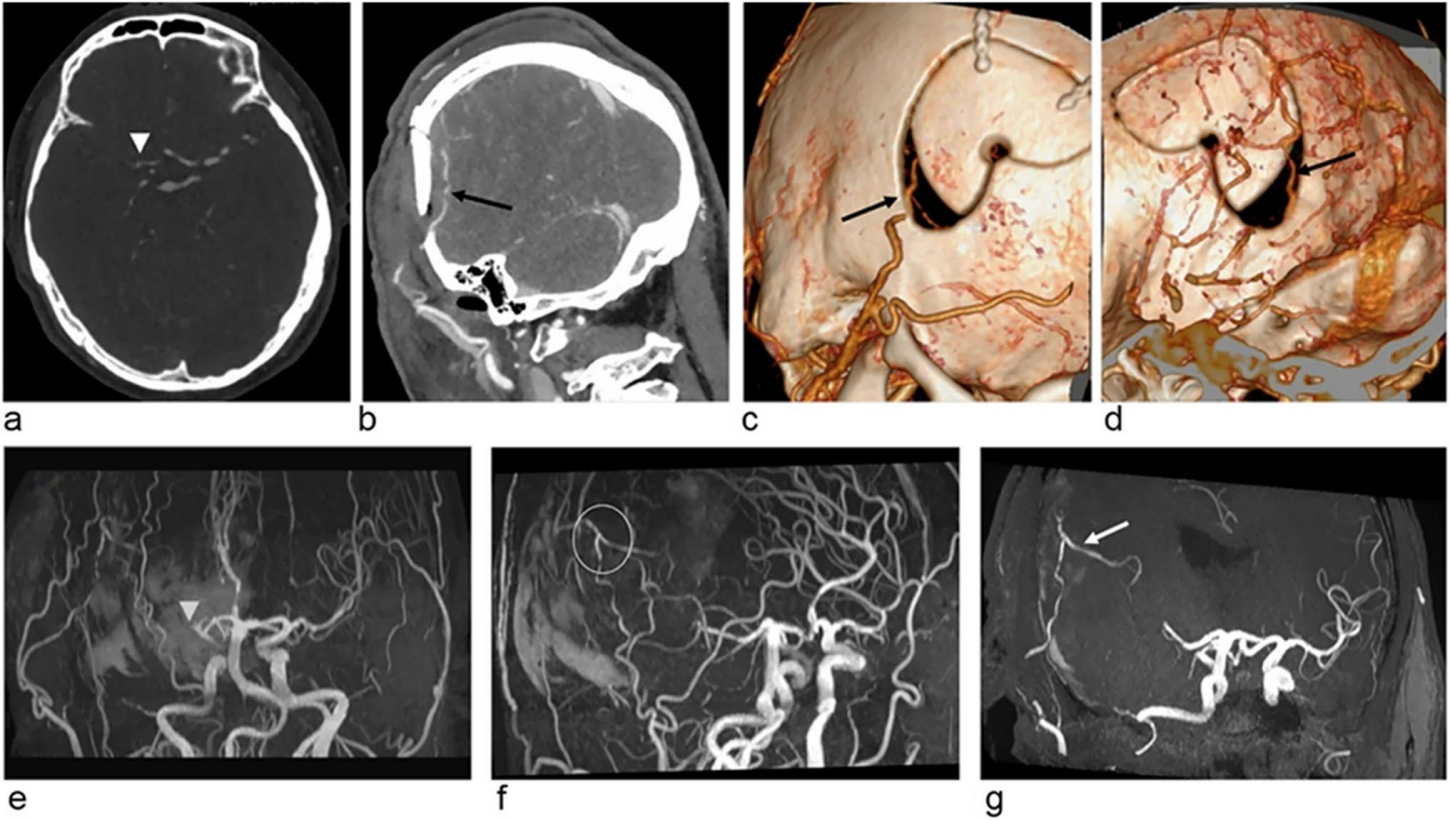
A 55-year-old patient underwent right-side STA-MCA bypass surgery. **a** Preoperative CTA and **e** preoperative MRA show the occlusion of the right MCA (arrowheads). **b, c, d** Postoperative CTA show that the right STA (black arrows) entered the cranium through the bone defect area of the temporal region. **f and g** Postoperative MRA clearly showed the anastomosis (circled area) and the distal branch of the MCA (white arrow). Image courtesy of Dr. Hanyu Jiang, Department of Radiology, West China Hospital

CTA can clearly show the circle of the Willis, as well as the anterior, middle, and posterior cerebral arteries and their main branches, providing an important diagnostic basis for occlusive vascular lesions. Because of its short acquisition time and fast image postprocessing, CTA is the first choice in emergency cases. In MMD, ischemic stroke can be diagnosed as early as 2 h after onset. Cortical surface imaging with CTA can be used to depict the number and distribution of MCA cortical arteries (M4) which are the main recipient arteries in STA-MCA bypass surgery [66]. Compared with MRA, CTA has the advantage to display the vascular status of the extracranial segment of STA (Fig. 2a–d) [35]. However, CTA has limitations in showing MMVs with small calibers (< 3 mm) and its resolution is lower than that of DSA. CTA may also be interfered by motion artifacts.

Dynamic 3D- and 4D-CTA can simultaneously display subtle angio-architectural features and vascular flow patterns, providing information on ongoing vascular changes as well as accurate spatial delineation of cerebrovascular pathologies [3]. However, the clinical practice of 4D-CTA is restricted by its high total radiation dose.

#### Computed tomography perfusion (CTP)

**Fig. 3:**
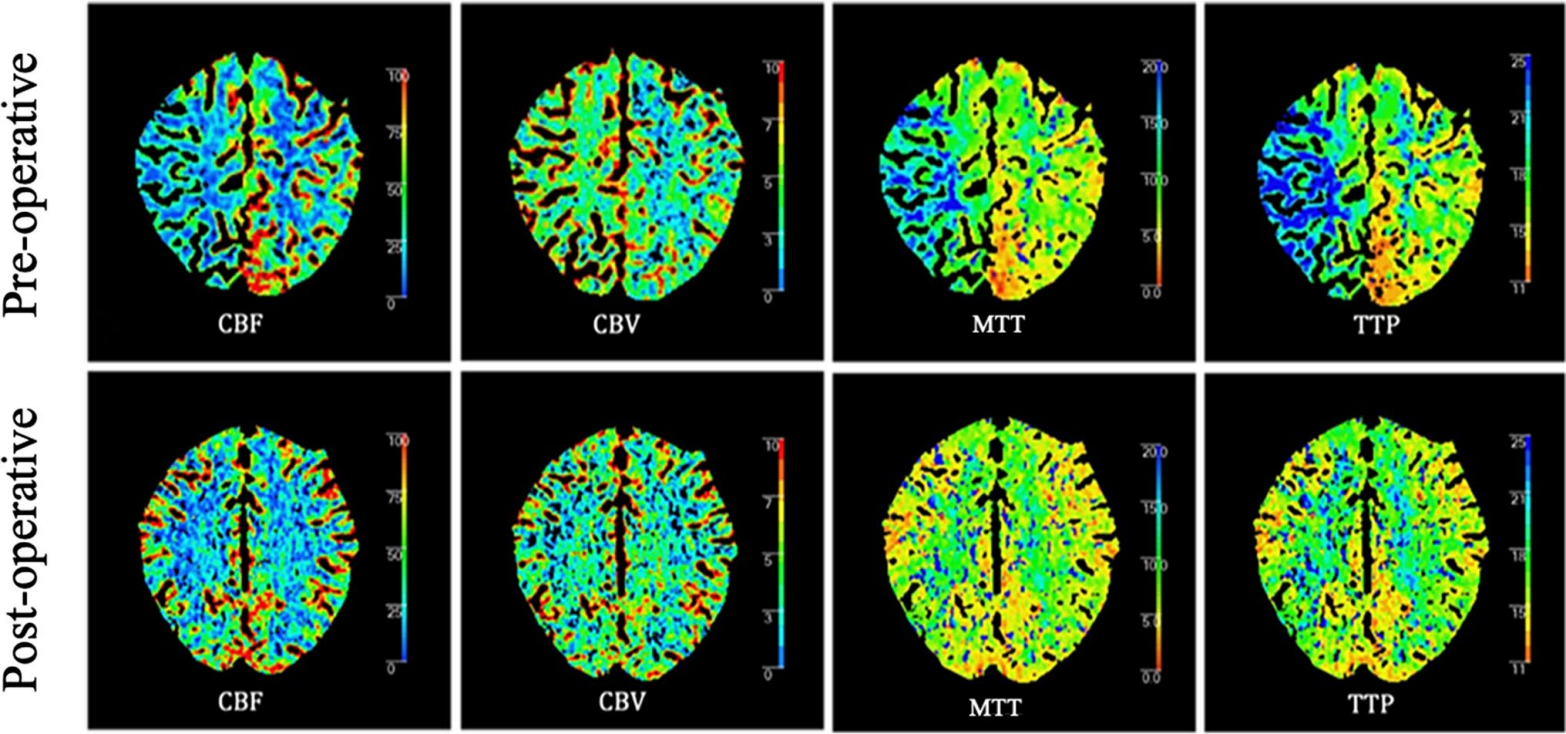
Hemodynamic changes of a 23-year-old female underwent STA-MCA bypass revascularization in the right hemisphere. Preoperative CTP (the upper row) shows that in the right MCA area, CBF was significantly decreased; MTT and TTP were significantly prolonged, while CBV was slightly increased. Six months after surgery, postoperative CTP (the bottom row) shows increased CBF, reduced CBV, shortened MTT and TTP in the same region. Image courtesy of Dr. Hanyu Jiang, Department of Radiology, West China Hospital

CTP has the advantages of short acquisition time and high spatial resolution, so it is generally the first choice to measure the changes of cerebral hemodynamics after surgery. Among the parameters extracted from the time intensity curve, the changes of TTP and MTT are the most sensitive ones in the early postoperative period [7], followed by CBF, which is also the matter of great concern to surgeons. CBF is closely related to bypass patency and can directly reflect the degree of blood supply recovery. The change in CBV is complicated, affected by the ability of different automatic regulation processes involving arterial, capillary, venous, and parenchymal components [10]. Previous studies have shown that in patients with ischemic MMD treated either with STA-MCA bypass surgery or surgery involving multiple burr holes, the postoperative MMT and TTP values are significantly shortened, CBF is significantly increased, while CBV might increase or decrease (Fig. 3) [7, 10, 65]. In a recent retrospective clinical study of 57 patients with hemorrhagic MMD, the CBV appeared to decrease and be relatively stable in the chronic phase after revascularization with varying degrees of MTT and TTP shortening, and no significant change in CBF [29]. Bypass surgery is of great value in the treatment of ischemic MMD, but it is still controversial in hemorrhagic MMD. Therefore, a larger sample size, multicenter prospective study with longer follow-up time is needed to confirm the difference in hemodynamic changes between the two stroke subtypes after bypass surgery.

### Magnetic resonance imaging

#### Magnetic resonance angiography (MRA)

MRA provides a noninvasive, radiation-free alternative to DSA and CTA for evaluating bypass patency and can be used to measure arterial caliber to predict the development of surgical collaterals [68]. Among MRA techniques, 3D TOF-MRA is the most commonly used technique for cerebral artery imaging with high spatial resolution and signal-to-noise ratio (SNR), as well as very thin slice thickness. In terms of showing the intracranial segment of the STA and EC-IC bypass anastomosis, TOF-MRA is at least as good as CTA or even better than CTA (Fig. 2e–f). However, as TOF-MRA is velocity-dependent, the turbulent flow at stenosis may cause phase dispersion and signal loss. It was shown that TOF-MRA is inferior to CTA in revealing trepanation bypass segments and overestimates focal pseudo-occlusive lesions in this area [6]. TOF-MRA also tends to overestimate ICA stenosis compared with contrast-enhanced MRA [71].

#### Intracranial vessel wall imaging (IVWI)

**Fig. 4:**
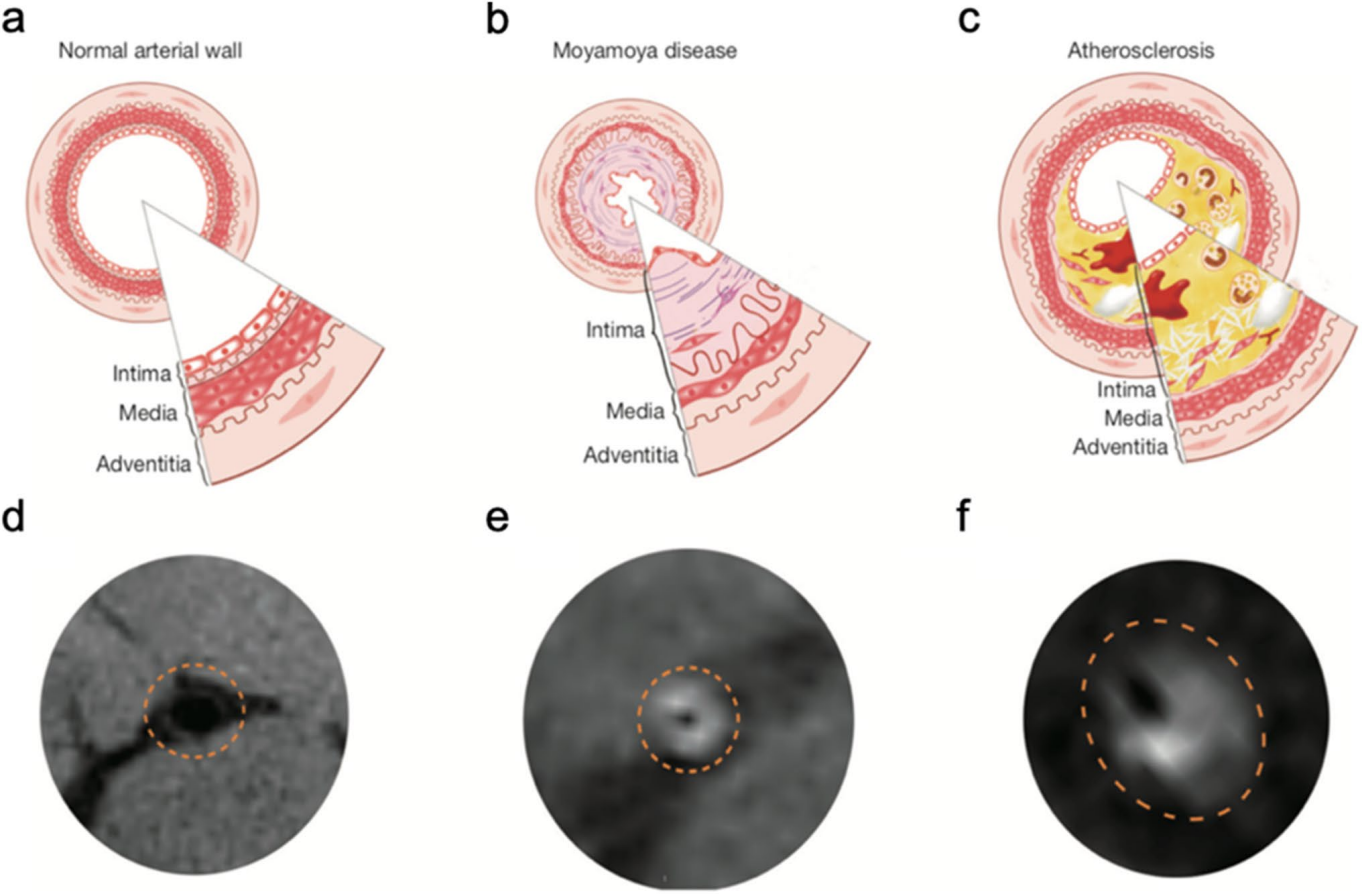
Pathological mechanism and IVWI manifestations of moyamoya disease and atherosclerosis. **a, d** Pathologic and IVWI findings of normal arterial lumens. **b, e** Compared with normal arterial wall, the affected artery wall in MMD is characterized by the thickness of intima and the atrophy of media, with homogeneous signal intensity and concentric vessel wall thickening on IVWI. **c, f** In atherosclerosis, the main etiology of arterial stenosis is the build-up of atherosclerotic plaque and the lipid deposition under the endothelium, with eccentric and heterogeneous vessel wall thickening on IVWI [73]

IVWI is an adjunct to conventional MRA and has great potential in morphological assessment of MMD revascularization. Based on high-resolution MRI (HR-MRI), IVWI has been proved to be effective to differentiate MMD from intracranial atherosclerotic stenosis (Fig. 4) [60, 73, 82]. On IVWI images, MMD tends to have homogeneous signal intensity enhancement and concentric vessel wall thickening, while intravascular atherosclerotic stenosis (IAS) shows eccentric and heterogeneous vessel wall thickening. Compared with IAS, the outer diameters of stenotic ICA and MCA are smaller in patients with MMD. High-grade arterial wall enhancement on IVWI may associate with the progression of angiopathy and a high risk of ischemic infarction [30, 58]. However, the difference in vessel wall enhancement between early and late angiographic stages of MMD remains to be further investigated [47]. More research is also needed to evaluate the value of IVWI in differentiating ischemic or hemorrhagic strokes caused by moyamoya disease.

#### Ultrahigh field intensity magnetic resonance

Although 1.5-T and 3.0-T MRI are commonly used, ultrahigh field (7.0-T) MRI is a promising neuroimaging technique for the evaluating of MMD especially the risk of hemorrhage. The advantages of TOF at 7.0-T include the increased SNR, longer T1 relaxation times augmenting the vessel-tissue contrast, and inherently hyperintense arterial vasculature at higher field strengths [34, 62, 63]. There was no significant difference between 3.0-T MRI and 7.0-T MRI in depicting the main intracranial arteries, but 7.0-T MRI could excellently delineate the collateral network pathways in MMD and is better correlated with Suzuki’s stage [12]. Susceptibility-weighted imaging (SWI) and TOF-MRA fusion images in 7.0-T MRI help to improve the detection of bleeding points in hemorrhagic MMD, and screening these patients will help to better assess the risk of intracranial hemorrhage [63]. At present, 7.0-T MRI has not been widely used in clinic, and more studies are needed to confirm its value in surgical reconstruction of hemorrhagic MMD.

#### Fluid-attenuated inversion-recovery MR imaging (FLAIR-MRI)

The “Ivy sign,” leptomeningeal high signal intensity, is an MMD-specific feature on fluid-attenuated inversion-recovery MR imaging, indicating slow flow of engorged pial vasculature toward leptomeningeal collaterals and “misery perfusion” areas. Studies showed that the decrease of the “ivy sign” on FLAIR-MRI after surgery is related to the improvement of cerebral hemodynamics and clinical symptoms, while de novo “ivy sign” may predict early postoperative CHS [22, 28, 31].

#### Dynamic susceptibility contrast MRI (DSC-MRI)

DSC-MRI is currently one of the most used MR perfusion imaging techniques to quantify cerebral hemodynamic changes by neurosurgeons. DSC-MRI can help to select candidates for MMD intervention and predict the outcome and risk of surgery. In the studies of Ishii et al. [24, 25], the MTT measured by DSC-MRI may depict the small amelioration of arterio-genesis as early as 2–4 weeks after indirect bypass surgery, far earlier than other imaging modalities. The degree of MTT delay shortening was positively correlated with the surgical effect. However, the disadvantages of DSC-MRI are the need for a contrast agent, insufficient quantitative reliability, and long resolution time [55].

#### Arterial spin labeling MRI (ASL-MRI)

**Fig. 5:**
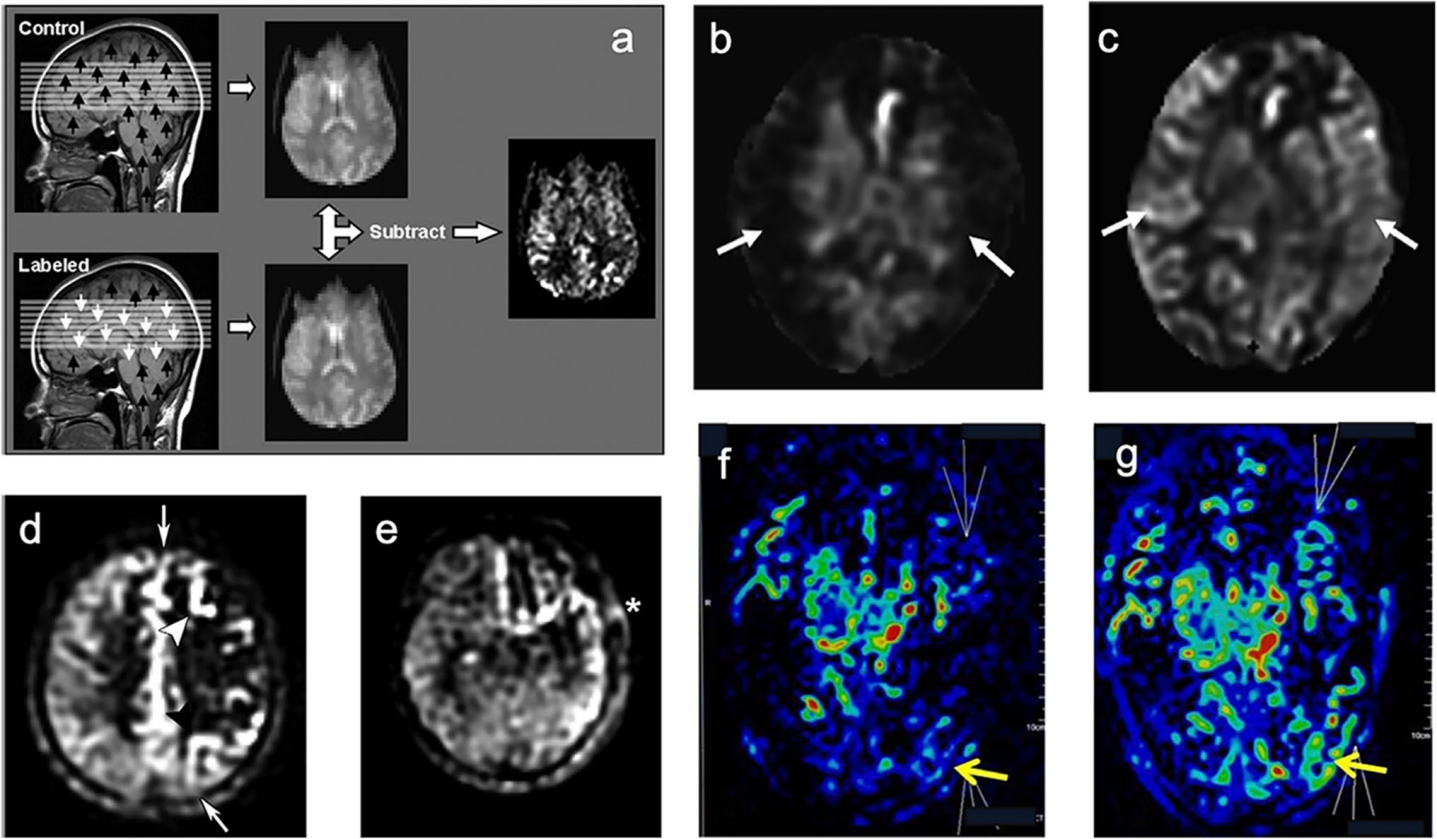
Arterial spin-labeled MRI in moyamoya disease. **a** Blood proton from the ICA was labeled once (labeled) and twice (controlled) by 180° radiofrequency pulse, respectively. After T1 delay, the protons arrived at the signal acquisition area, and the labeled image and control image were obtained respectively. After subtraction of these two images, a difference image with intensity proportional to cerebral blood flow (CBF) was obtained, i.e., cerebral perfusion image. **b, c** ASL sequences of a moyamoya patient with good recovery after bilateral bypass surgery. The preoperative ASL (**b**) signal (*arrows*) war dark, indicating markedly reduced perfusion to bilateral MCA territories. The postoperative image (**c**) shows increased ASL signal in the bilateral MCA territories (*arrows*). **d, e** ASL sequences of a moyamoya patient with poor recovery after left bypass surgery. Preoperative ASL images (**d**) show decreased perfusion (arrows) and arterial transit artifact (ATA) signals in left distal ICA territories (arrowheads) indicating late-arriving flow via collateral pathways. After left MCA–STA bypass surgery, postoperative ASL images (**e**) still show impaired perfusion and ATA signals in left MCA territory and prominent ATA signal in MCA–STA anastomosis site ( ∗) indicating flow stagnation in anastomosis site. **f, g** Color-coded ASL perfusion images of a moyamoya patient who had moderate to severe headache with seizures at the second postoperative day, and was clinically suspected as hyperperfusion syndrome. There was a significantly raised perfusion (more than 100% increase in CBF values) in the left occipital regions (yellow arrows) between preoperative (**f**) and postoperative (**g**) [39, 49, 56, 72]

ASL-MRI uses magnetically labeled inflowing blood as an endogenous tracer to estimate brain perfusion at the tissue level [72, 83], especially suitable for pediatric patients with MMD [5, 18, 56]. Pulsed arterial spin labeling (PASL), continuous arterial spin labeling (CASL), and pseudo-continuous arterial spin labeling (pCASL) are three labeling methods, among which pCASL is the most superior based on high labeling efficacy and signal to noise. Recent studies suggest that ASL-MRI is well correlated with DSC-MRI, CTP, PET, and SPECT in mapping CVR and CBF [16, 17, 51, 52, 70, 83]. Studies have demonstrated the clinical significance of ASL-MRI in evaluation postoperative hemodynamic dysfunction [18, 49]. Compared with preoperative cerebral perfusion, there was no significant increase in ASL signal in the bypass area, suggesting that there was still hypoperfusion. When ASL signal increases more than 100%, researchers should be alert to the risk of CHS (Fig. 5).

However, ASL-MRI has an inherent deficiency, caused by its imaging mechanism. For instance, the measured CBF could be underestimated due to the long arterial transit times (ATTs) in steno-occlusive arterial segments and collateral pathways. Under these circumstances, the use of multi-delay or long-label-long-delay algorithms in ASL-MRI could help improve the accuracy of CBF assessments with satisfactory SNR [13, 70]. Interestingly, high-intensity signals of arterial transit artifacts (ATA) caused by ATTs could be in turn, used to depict the collateral flow in morphology [39, 84]. Overall, ASL-MRI is a promising noninvasive alternative for evaluating the hemodynamics of MMD when nuclear medical imaging methods are not available, but its limitations such as weak signal intensity, low image resolution, and poor repeatability limit it from becoming a standalone imaging method for clinical cerebral perfusion assessments.

#### Blood oxygen level-dependent functional MRI (BOLD-fMRI)

BOLD-fMRI is a brain mapping technique using deoxyhemoglobin in the blood vessels as an endogenous contrast agent to produce functional activation maps. BOLD-fMRI is widely used to monitor CVR and neurovascular coupling changes and assess surgical efficacy following revascularization [59, 61]. Previous BOLD-fMRI studies often change the concentration of end-expiratory carbon dioxide to map CVR with breath-holding or CO_2_ inhalation techniques [11, 20]. Liu et al. [41] proposed using resting-state BOLD data to map CVR, which can avoid gas inhalation or breath-holding. The global BOLD signal in the frequency range of 0.02–0.04 Hz provides the best estimation of the spontaneous fluctuation of blood CO_2_ concentration, relative to that in other frequency bands. BOLD-fMRI holds future potential in becoming a routine examination in the pre- and post-operative evaluation of MMD patients especially for pediatric patients. However, how to improve the stability and repeatability of BOLD imaging is a major concern in clinical practice.

### Nuclear medical imaging techniques

#### Single-photon emission computed tomography (SPECT)

SPECT is considered the reference standard technique for CBF perfusion assessments [16, 64]. As radioactive tracers, ^99^mTc-ECD, ^99^mTc-HMPAO, and ^123^I-IMP can enter into brain cells through the normal blood–brain barrier and can be used to assess the regional brain function. A hypo-responsive CVR on postoperative basal or acetazolamide-challenged SPECT in patients with MMD suggests a poor prognosis such as remaining neurological deficits and ischemic attacks during follow-up [65]. Association of cognitive dysfunction with improvement with CBF changes measured by SPECT has been explored both in adult and pediatric patients with MMD [32, 69].

Yanagihara et al. [74] recently found that CHS in the acute stage after bypass surgery impairs cognitive function and that an increase in CBF in the chronic stage improves cognitive function in adult patients with symptomatic ischemic MMD and misery perfusion.

#### Positron emission tomography (PET)

PET is considered the standard functional imaging method for quantifying metabolic processes that are relevant to MMD vascular functionalities after bypass surgery. H_2_^15^O, ^15^O_2_, and C^15^O_2_ are commonly used as tracers in PET for cerebral hemodynamic assessments. The oxygen extraction fraction (OEF) and cerebral metabolic rate of oxygen (CMRO_2_) are important parameters for making treatment decisions in MMD and are closely related to CVRC, hemodynamic stress distribution, and characteristic features of collateralization [23]. H_2_^15^O PET enabled detection of impaired CVRC in a large number of symptomatic MMD patients who had negative findings on ^99^mTc-HMPAO SPECT in Acker’s study [1]. Kaku et al. [27] revealed that symptomatic CHS in patients with MMD could be characterized by temporary increases in CBF > 100% over the preoperative values, and among the preoperative PET parameters, increased OEF was the only significant risk factor for CHS. Recently, H_2_^15^O PET has been used for the preoperative assessment of neuropsychological impairment by measuring territorial CVR, and ^15^O PET has been used to investigate the improvement in cognitive decline by measuring changes in OEF and CMRO_2_ following indirect bypass surgery [19, 57]. These advances will be more conducive to obtaining more accurate information through PET imaging to select MMD patients who can benefit the most from surgery. However, PET is limited by its general clinical unavailability, high cost, and lengthy measurement time.

### Fluorescence imaging

**Fig. 6:**
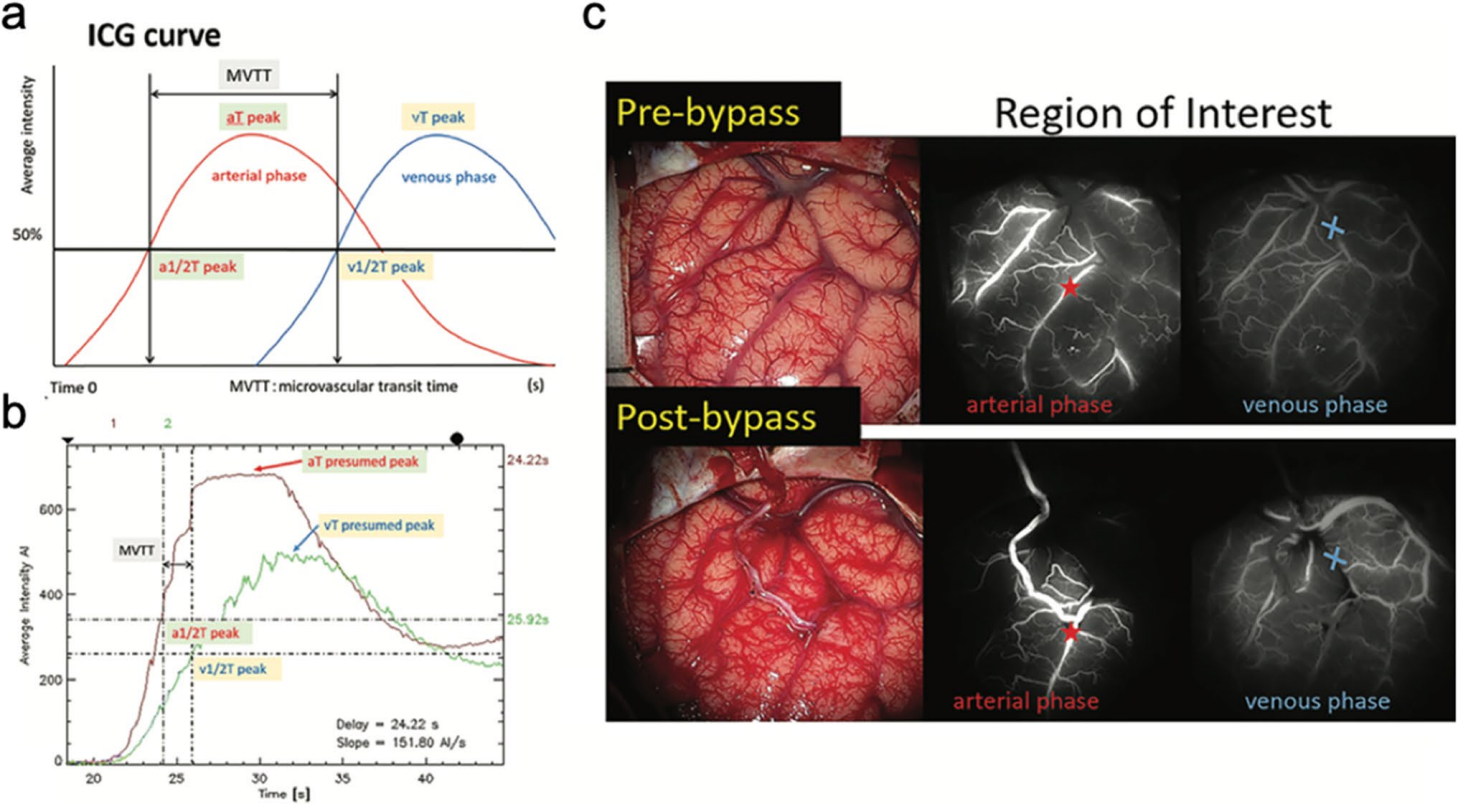
Morphological visualization and quantitative assessment of STA-MCA bypass in MMD by ICG with FLOW 800 software. **a** Microvascular transit time (MVTT) was calculated as venous T_1/2_ peak–arterial T_1/2_ from ICG time intensity curve. **b** The “time to half-value of peak” (T_1/2_ peak) was used instead of “time to peak,” because the “time to peak” was difficult to define. **c** Analysis of the ICG time intensity curve was performed on the same ROIs in arterial phase (asterisk) and venous phase (cross) respectively before and after bypass surgery [75]

Indocyanine green and sodium fluorescein are commonly used imaging agents for intraoperative fluorescence imaging. With the help of Flow Insight software® (Carl Zeiss, Co.) or Flow 800 software, the direction of blood flow can be “visualized.” The direction of blood flow after bypass is related to the pressure difference between donor and recipient vessels, and the watershed migration can occur, suggesting the dysfunction of the cortical cerebral hemodynamics. Intraoperative fluorescence imaging provides semiquantitative regional hemodynamic alterations and can be used to identify patients at high risk of transient neurological events; thus, the perioperative treatment can be adjusted (Fig. 6) [69, 75, 76]. Horie et al. [21] found that the mismatch ratio of donor STA/recipient MCA and poor run-off or stagnation of blood flow from the STA might contribute to postoperative CHS in patients with MMD.

Another similar optical imaging technique is microscopic cortical venous redness (venous reddening). This digital photographic method is based on intraoperative measurement of venous oxygen saturation by venous R intensity, which can indirectly represent tissue oxygen metabolism and CBF impairment, and is of great value in detection of CHS immediately after anastomosis [45, 46].

### Ultrasonography (US)

#### Transcranial color-coded duplex sonography (TCCS)

**Fig. 7:**
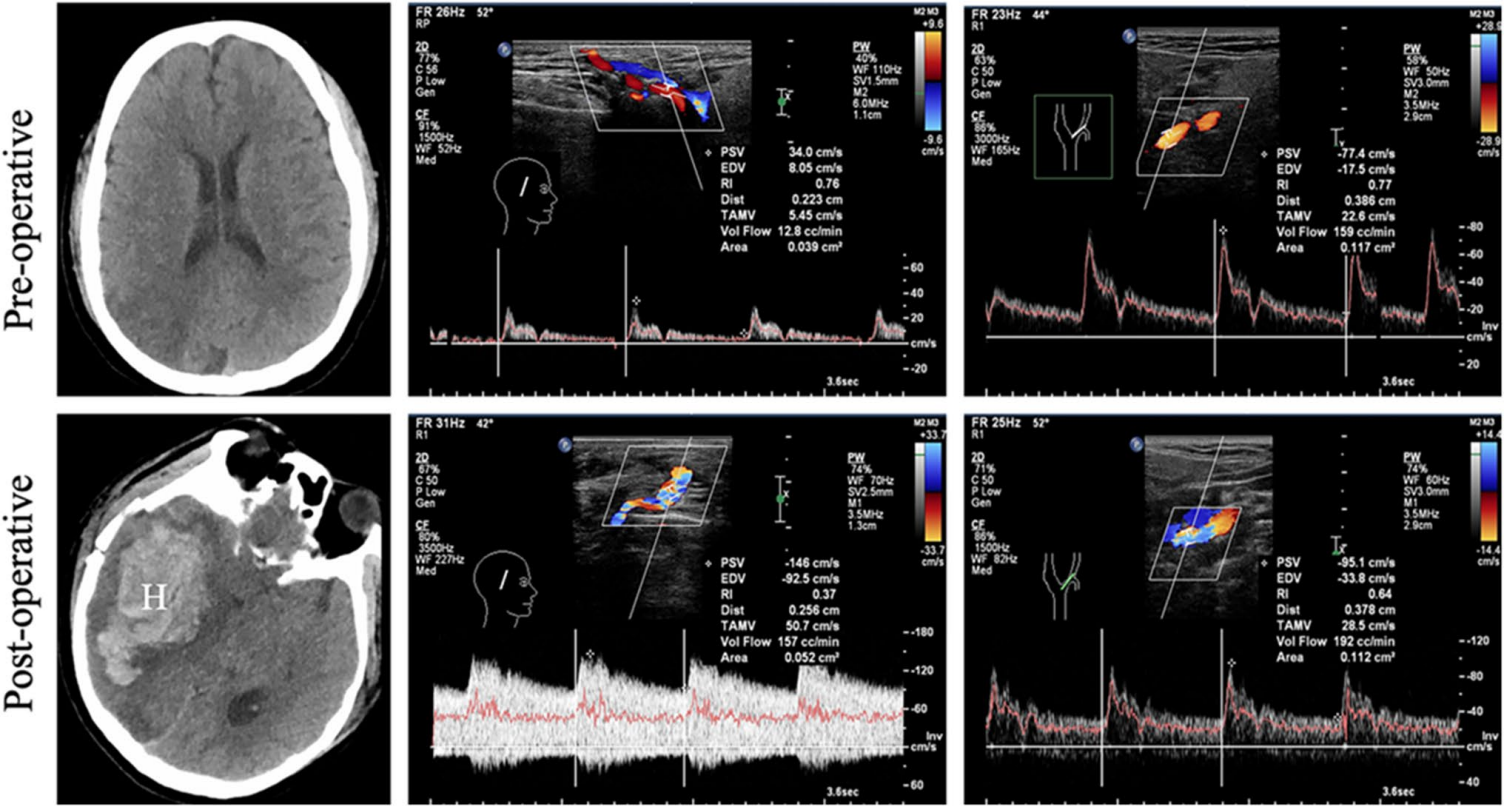
A 44-year-old male MMD patient underwent right-side STA-MCA bypass surgery. One week after, the patient was found CHS manifesting with headache, vomiting, and consciousness disturbance. Emergency CT showed a large hematoma in the right frontotemporal insular lobe, complicated with basal ganglia hemorrhage and cerebral hernia (the left column). On TCCS, PSV at the proximal end of STA increased sharply more than 4 folds, RI decreased by half (the middle column), and the flow velocity of ECA slightly increased (the right column). H indicates hematoma; CHS, cerebral hyper-perfusion syndrome. Image courtesy of Dr. Chenyun Zhou, Department of Ultrasound, West China Hospital

TCCS can provide in real-time quantitative information on donor and recipient arteries, which is an ideal imaging tool to monitor graft flow postoperatively [77, 79]. Quantitative parameters such as peak-systolic velocity (PSV), end-diastolic velocity (EDV), mean flow volume (MFV), pulsatility index (PI), and resistance index (RI) are usually measured through the trans-temporal, trans-orbital, and trans-foraminal windows [2]. The increases of PSV and EDV or the decreases of RI and PI of the STA indicate that there is blood supply in an extracranial-to-intracranial direction. In addition, the changes in EDV in the STA or ECA were well correlated with Matsushima grading system of MMD [78]. The imbalance between the graft flow and the MCA network may result in postoperative cerebral hyper-perfusion (CHS). At our hospital, CHS was detected by TCCS in a male patient who underwent STA-MCA bypass on postoperative day 10 during follow-up. The US findings showed more than 3 folds of increase in PSV, and a significantly decreased of RI in the STA (Fig. 7).

#### Intraoperative US (IVUS)

For intraoperative IVUS, a perivascular flow probe can be precisely positioned over the freely dissected target vessel without attenuation of the acoustic beam, which allows quickly detecting graft patency and revising problematic grafts, thus greatly improving the success rate of the operation [38, 80, 81]. Compared with ICG-VAG, micro-Doppler imaging can not only help to effectively identify recipient artery before anastomosis effectively, but also help to check the flow velocity and direction of blood flow after bypass surgery, characterizing hemodynamic status [48, 53].

#### Transcranial Doppler (TCD)

TCD is a commonly used clinical tool for monitoring intracranial ischemic diseases in neurology department because of its characteristics of non-invasive, quick, bedside, and cheap. Cerebral vasomotor reactivity (VMR) measured by TCD can reflect cerebrovascular reserve, providing an important reference for preoperative hemodynamic assessment [26]. In cases of stenosis of terminal ICA, TCD showed abnormally elevated blood flow velocity and increased PI at the stenosis site, presenting eddy current spectrum. When an artery occlusion occurs, the flow velocity is slow and the spectrum changes into a wavy pattern. In the late stage of moyamoya disease, the blood flow velocity of ECA and its branches increased significantly, and the morphology of intracranial blood flow spectrum changed accordingly.

#### Contrast-enhanced ultrasound (CEUS)

CEUS is a promising imaging technique for visualizing microvascular circulation and brain perfusion with qualitative and semi-quantitative information. CEUS has been used to assess neurosurgical conditions such as intracranial tumors, arteriovenous malformations, and aneurysms [33, 36]. The application of CEUS in evaluating MMD revascularization is not well known to neurosurgeons. In a recent rat model of MCA occlusion, transcranial CEUS revealed striking decreases in cortical and striatal blood volume, flow velocity, and cerebral perfusion during ischemic stroke. After vessel recanalization, blood volume and perfusion increased twofold above the baseline value, which is indicative of acute CHS [54]. This study mimics the mechanism of surgical bypass of MMD, which lays a foundation for the future intraoperative CEUS evaluations in human MMD.

## Discussion

Moyamoya disease is a potential risk factor for stroke. Complex vascular remodeling, whether due to disease progression or surgical intervention, leads to changes in cerebral morphology, hemodynamics, and clinical improvements or complications. Early detection of these changes and their concomitant effects is the key to the treatment of moyamoya disease. Table 1 compares and discusses the main roles and applicable scenarios of each imaging modality in revascularization of moyamoya disease. In pre-operative period, DSA is still considered the gold standard for diagnosis and grading up to now; however, in pediatric patients or patients with generally poor conditions, MRA or CTA may be an alternative. In intra-operative period, fluorescence imaging and IVUS are superior in real-time monitoring of blood flow in a certain artery. It is recommended that regular hemodynamic assessment by TCCS examination should be performed regularly at 1 week, 1 month, 3 months, 6 months, and 1 year after surgery, and CTP examination should be carried out half a year after the operation. Patients with unilateral moyamoya disease should undergo CTA or MRA once a year to evaluate disease progression. DSA could be carried out if there are new neurological symptoms. SPECT and PET are not currently necessary because they are expensive but can be used conditionally.

**Table 1:** Comparison of imaging modalities used in revascularization of moyamoya disease

Imaging modality	Main roles in MMD revascularization	Hemodynamic evaluation (Quantitative parameters)	Applicable scenario			Contrast materials and Tracers
			Pre-op	Intra-op	Post-op	
DSA	•Gold standard for MMD diagnosis and grading [44] •Proof of patency of EC-IC bypass [15] •Presentation of the collateral circulation [14, 43] •Identification of concomitant diseases [4, 42, 50]	MTT, TTP, AUC	** + + + **	** + **	** + + **	Iodinated contrast agents
CT	• Emergency situations •Visualization of abnormal basal vascular network and MCA cortical arteries [66] •Evaluation of postoperative cerebral perfusion recovery [7, 10, 29, 65]	CBV, CBF, MTT, TTP	** + + + **	** + **	** + + + **	Iodinated contrast agents
MRI	•Monitoring of intracranial artery stenosis and ischemic changes [27] •Intracranial vessel wall imaging [60, 73, 82] •Better display of MMVs on ultrahigh field intensity MRI [12, 34, 62, 63] •Evaluation of postoperative hemodynamic recovery or dysfunction [18, 24, 25, 49] •Assessment of surgical efficacy and prediction of patients’ prognosis [59, 61]	CBV, CBF, MTT, TTP, CVR	** + + + **	** + **	** + + + **	Exogenous material: Gadolinium chelate (DSC-MRI); Endogenous material: Arterial water proton (ASL-MRI), Deoxyhemoglobin (BOLD-fMRI)
SPECT	•The reference standard for cerebral perfusion [16, 64]	CBF, CBV, CVR	** + + **	**-**	** + + **	^99^mTc-HMPAO, ^99^mTc-ECD, ^123^I-IMP
PET	•Metabolic assessment of brain tissue [23] •Assessment of neuropsychological impairment and recovery [19, 57]	CBV, CBF, OEF, CMRO_2_, CVRC	** + + **	**-**	** + + **	^15^O_2_, C^15^O_2_, H_2_^15^O
Fluorescence imaging	•Real-time imaging of bypass, and direct monitoring of blood flow [69, 75, 76]	MTT, TTP, AUC	**-**	** + + + **	**-**	Indocyanine green, Sodium fluorescein
US	•Quantification of blood flow of donor, recipient, and bypass arteries [2, 77–79] •Intraoperative navigation [48, 53] •Cerebral vasomotor reactivity assessment [26] •Mapping of cerebral vascular circulation [54]	PSV, EDV, PI, RI	** + + **	** + + + **	** + + + **	None (TCCS, TCD) Microbubbles(CEUS)

*DSA*, digital subtraction angiography; *CT*, computed tomography; *MRI*, magnetic resonance imaging, *ICG-VAG*, indocyanine green video-angiography; *US*, ultrasonography; *SPECT*, single-photon emission computed tomography; *PET*, positron emission tomography; *TCCS*, transcranial color-coded duplex sonography; *TCD*, transcranial Doppler; *CEUS*, contrast-enhanced ultrasound. *Pre-op*, preoperative; *Intra-op*, intraoperative; *Post-op*, postoperative; *AUC*, area under the curve; *CVR*, cerebral vascular reserve; *CVRC*, cerebrovascular reserve capacity; *OEF*, oxygen extraction fraction; *CMRO*_*2*_, cerebral metabolic rate of oxygen; *MTT*, mean transit time; *TTP*, time to peak; *AUC*, area under the curve; *PSV*, peak-systolic velocity; *EDV*, end-diastolic velocity; *PI*, pulsatility index; *RI*, resistance index

“ +  +  + ” highly recommended; “ +  + ” recommended; “ + ” moderately recommended; “-” less recommended

## Summary

Neuroimaging can assist surgeons to optimize matching selection of donor-recipient arteries, prevent the occurrence of postoperative perfusion disorders, and improve the accuracy of bypass as much as possible. In recent years, the rapid development of imaging technology, such as MRI and ultrasound, with their advantages of radiation-free, fast, and low cost, plays an increasingly important role in the diagnosis and treatment of moyamoya disease, especially in pediatrics.

## Data Availability

Not applicable.

## Abbreviations

MMD: Moyamoya disease
MMVs: Moyamoya vessels
IAS: Atherosclerotic stenosis
CHS: Cerebral hyper-perfusion syndrome
ICA: Internal carotid artery
ECA: External carotid artery
STA: Superficial temporal artery
MCA: Middle cerebral artery
PCA: Posterior cerebral artery
EC-IC: Extracranial-intracranial
CBF: Cerebral blood flow
CBV: Cerebral blood volume
MTT: Mean transit time
TTP: Time to peak
PSV: Peak-systolic velocity
EDV: End-diastolic velocity
MFV: Mean flow volume
RI: Resistance index
CVR: Cerebrovascular reserve
CVRC: Cerebrovascular reserve capacity
OEF: Oxygen extraction fraction
CMRO_2_: Cerebral metabolic rate of oxygen
IVWI: Intracranial vessel wall imaging
DSC-MRI: Dynamic susceptibility contrast MRI
ASL-MRI: Arterial spin labeling MRI
BOLD-fMRI: Blood oxygen level-dependent functional MRI
FLAIR-MRI: Fluid-attenuated inversion-recovery MR imaging
SPECT: Single-photon emission computed tomography
PET: Positron emission tomography
ICG-VAG: Indocyanine green video-angiography
TCCS: Transcranial color-coded duplex sonography
TCD: Transcranial Doppler
CEUS: Contrast-enhanced ultrasound
